# Development of Composite, Reinforced, Highly Drug-Loaded Pharmaceutical Printlets Manufactured by Selective Laser Sintering—In Search of Relevant Excipients for Pharmaceutical 3D Printing

**DOI:** 10.3390/ma15062142

**Published:** 2022-03-14

**Authors:** Piotr Kulinowski, Piotr Malczewski, Marta Łaszcz, Ewelina Baran, Bartłomiej Milanowski, Mateusz Kuprianowicz, Przemysław Dorożyński

**Affiliations:** 1 Institute of Technology, Pedagogical University of Cracow, Podchorążych 2, 30-084 Cracow, Poland; piotr.kulinowski@up.krakow.pl (P.K.); pmalczewski@gmail.com (P.M.); ewelina.baran@up.krakow.pl (E.B.); 2 Department of Falsified Medicines and Medical Devices, National Medicines Institute, Chełmska 30/34, 00-725 Warsaw, Poland; m.laszcz@nil.gov.pl; 3 Chair and Department of Pharmaceutical Technology, Poznan University of Medical Sciences, ul. Grunwaldzka 6, 60-780 Poznan, Poland; b.milanowski@rcz-zbaszyn.pl; 4 GENERICA Pharmaceutical Lab, Regionalne Centrum Zdrowia Sp. z o.o., Na Kępie 3, 64-360 Zbąszyń, Poland; m.kuprianowicz@rcz-zbaszyn.pl; 5 Department of Drug Technology and Pharmaceutical Biotechnology, Medical University of Warsaw, Banacha 1, 02-097 Warsaw, Poland; 6 Department of Spectroscopic Methods, National Medicines Institute, Chełmska 30/34, 00-725 Warsaw, Poland

**Keywords:** pharmaceutical additive manufacturing, powder bed fusion, composite materials, drug delivery, 3D printing, gastroretentive drug delivery systems, floating dosage forms, nylon, polyamide 12, metronidazole

## Abstract

3D printing by selective laser sintering (SLS) of high-dose drug delivery systems using pure brittle crystalline active pharmaceutical ingredients (API) is possible but impractical. Currently used pharmaceutical grade excipients, including polymers, are primarily designed for powder compression, ensuring good mechanical properties. Using these excipients for SLS usually leads to poor mechanical properties of printed tablets (printlets). Composite printlets consisting of sintered carbon-stained polyamide (PA12) and metronidazole (Met) were manufactured by SLS to overcome the issue. The printlets were characterized using DSC and IR spectroscopy together with an assessment of mechanical properties. Functional properties of the printlets, i.e., drug release in USP3 and USP4 apparatus together with flotation assessment, were evaluated. The printlets contained 80 to 90% of Met (therapeutic dose ca. 600 mg), had hardness above 40 N (comparable with compressed tablets) and were of good quality with internal porous structure, which assured flotation. The thermal stability of the composite material and the identity of its constituents were confirmed. Elastic PA12 mesh maintained the shape and structure of the printlets during drug dissolution and flotation. Laser speed and the addition of an osmotic agent in low content influenced drug release virtually not changing composition of the printlet; time to release 80% of Met varied from 0.5 to 5 h. Composite printlets consisting of elastic insoluble PA12 mesh filled with high content of crystalline Met were manufactured by 3D SLS printing. Dissolution modification by the addition of an osmotic agent was demonstrated. The study shows the need to define the requirements for excipients dedicated to 3D printing and to search for appropriate materials for this purpose.

## 1. Introduction

Application of Floating Drug Delivery Systems (FDDS) is the primary method of increasing gastric residence time [1]. Therefore, FDDS are suitable for the treatment of local *Helicobacter pylori* gastrointestinal infections [2]. Metronidazole (Met) belongs to the group of first choice chemotherapeutics in *H. pylori* treatment. Its pKa is 2.6 and its solubility in diluted (0.1 mol/L) hydrochloric acid is 2.2 mg/mL [3,4]. The study of Shah et al., 1999 has shown that Met is stable in simulated gastric fluid (SGF, pH 1.2) at 37 °C for 24 h [5]. 

Various technologies are currently used to develop FDDS [6,7]. In the last decade, additive manufacturing techniques (3D printing) have been applied to produce drug delivery formulations [8,9,10,11,12,13,14]. 3D printing is a promising technology for manufacturing controlled-release drug delivery systems, especially those that need additional features, e.g., floating properties. Several attempts have been made to develop the FDDS using additive manufacturing techniques in recent years. The main methods applied in the studies are fused deposition modeling [15] or semisolid extrusion [16,17,18]. 3D printing has also been used to prepare devices for maintaining the buoyancy of standard tablets [19,20,21,22] or to develop formulations with shells or hollow chambers that allow them to float [17,18,23,24,25]. 

Among various 3D printing techniques used to manufacture drug delivery systems, the application of Selective Laser Sintering (SLS) is relatively rare [26,27,28,29]. However, the possibility to manufacture various oral drug delivery systems, including orodispersible [30,31,32], immediate- [33] and modified-release [33,34,35,36] formulations, has been presented. According to the American Society for Testing Materials, SLS is classified as a Powder Bed Fusion (PBF) method of building objects by sintering powder particles using laser energy [27]. One of the most important advantages of the SLS technique is that it uses powders. Technologies for powder preparation and processing are well-established in the pharmaceutical industry. Once having the powder’s basis set, only powder calibrating and mixing are needed. Such an approach could simplify the preparation of particular formulations immediately before the printing process, making them applicable on the small manufacturing scale, e.g., hospitals or community pharmacies [29]. 

Currently available pharmaceutical-grade materials are designed primarily for tableting. The good mechanical properties of traditional drug delivery systems are obtained by powder compression in the tableting process. In this case, specific excipient properties are expected (e.g., tabletability, compressibility, elasticity and disintegration ability). Excipients optimized for powder compression technology cannot give similar results when using 3D printing due to different characteristics of the manufacturing process. In the case of SLS, the optical, thermal and rheological properties should also be considered [37]. Therefore, the use of new manufacturing techniques raises the problem of finding appropriate excipients.

The materials of choice for SLS are aliphatic polyamides (nylons). They are a group of synthetic polymers with favorable physicochemical properties, such as thermal stability combined with thermoplasticity, mechanical strength, chemical inertness, hydrophilicity and a high purity level after synthesis. The extensive clinical use of nylons as surgical materials together with other biomedical applications, including wound healing, manufacturing of dental implants, regenerative medicine and drug delivery, confirms their biocompatibility and non-toxicity [38]. These properties allow nylons to be used to formulate sustained-release drug delivery systems. Nylon 6,10 has been used to prepare erodible and monolithic matrix tablets with amitriptyline [39,40]. Nylon drug delivery systems have been proposed early in developing 3D printed pharmaceutical products using SLS. Unfortunately, prolonged drug release up to 7–9 days has been obtained, which is useless when dealing with oral dosage forms [41]. 

Another issue that remains to be solved is the high-dose API incorporation in printed drug delivery systems. SLS of highly drug-loaded controlled release matrices consisting of crystalline drug and no more than 5% of excipients has been proposed [36]. The extension of the presented concepts could be the use of the materials recommended for selective laser sintering, e.g., nylon, to create the insoluble mesh with the active pharmaceutical ingredient (API) embedded in the structure of the matrix. Nylon 12 (PA12) is a semi-crystalline thermoplastic polymer widely used for SLS, as commercial stained PA12 powders are available. In the current study, it was decided to use PA12 as an excipient for sintering matrix tablets containing Met. The study’s goals were: (1) to present the proof of concept of obtaining a highly drug-loaded, polymer-reinforced composite formulation, using FDDS as a working example; (2) to evaluate the properties of nylon as an inert 3D SLS printable excipient for Met incorporation; (3) to investigate the extended in vitro drug release behavior and to assess possible degrees of freedom for dissolution tuning.

## 2. Materials and Methods

Metronidazole (Hubei Hongyuan Pharmaceutical Technology Co., Ltd. Fengshan, China), commercial, carbon-stained nylon 12 for 3D printing (PA12 Powder, Sintratec AG, Brugg, Switzerland) and sodium chloride (Merck KGaA, Darmstadt, Germany) were used in the study. All other applied materials were of analytical grade.

### 2.1. 3D Printing 

Initially homogenized through sieve 160 mesh, powders of fine, pure crystals of Met, PA12 or sodium chloride (optional) were placed volumetrically (without tapping) in the glass measuring cylinder (1 L). The measured volumes were then weighed. The mixtures were prepared by mixing volumetric ratios of components in a polyethylene bag for 5 min. The volumetric and corresponding weight compositions of the printed tablets (printlets) are presented in Table 1.

**Figure 1:**
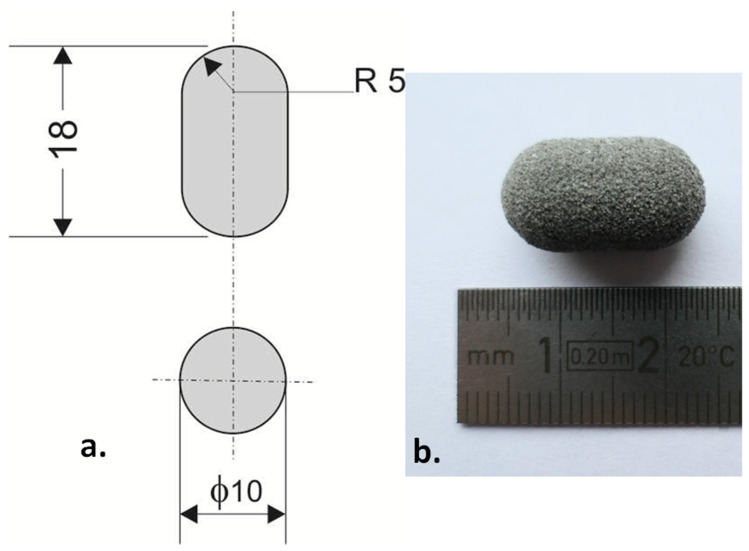
The example of printlet: (**a**) technical drawing (dimensions in mm), (**b**) printed Formulation A (80/20/0/100).

The capsule-shaped templates for printing were prepared in STL format with Autodesk Inventor 2020 (Autodesk Inc., San Rafael, CA, USA). The printlets were 1.8 cm long, and the diameter of the capsules was 1 cm (Figure 1a).

SLS was performed using Sintratec Kit 3D (Sintratec AG, Brugg, Switzerland) 3D printer equipped with a blue laser (2.3 W, 445 nm wavelength), operating under Sintratec Central software (v. 1.2.5, Sintratec AG, Brugg, Switzerland). The initial printing parameters were tuned experimentally, and the following temperature targets were applied: powder surface temperature 115 °C and chamber temperature 100 °C. The thickness of a powder layer was 150 μm, and the distance between two consecutive laser scans in the layer (hatch spacing) was 250 μm for all formulations. Laser speed was in the range of 75–150 mm/s depending on the particular formulation, according to Table 1. Nine printlets per print job were manufactured. 

### 2.2. Scanning Electron Microscopy (SEM)

The powder microstructure and surface of printed samples were observed using a scanning electron microscope JSM-6610LV (JEOL GmbH, Tokyo, Japan). The tests were conducted using a low vacuum with a backscattered electron-composition image (BEC). The accelerating voltage was 15 kV. The chemical analysis was performed with energy dispersion spectroscopy (EDS) using the X-Max detector, equipped with Aztec v.2.1 software (Oxford Instruments, Oxford, UK).

### 2.3. Differential Scanning Calorimetry (DSC)

Differential scanning calorimetry measurements were performed using the DSC822e cell with IntraCooler and STARe software v.8.10 (Mettler-Toledo, Columbus, OH, USA). Samples were weighed as received without preparation. About 10 mg of a studied sample was weighed into the standard aluminum crucible (40 µL). The crucible was hermetically sealed, and a lid was perforated before measurement. The samples were heated from 25 °C to 200 °C at 10 °C/min under a nitrogen atmosphere with 60 mL/min flow. The following temperature program was also used: (1) heating from 25 °C to 200 °C at 10 °C/min; (2) 5 min at 200 °C; (3) cooling from 200 °C to −60 °C at 50 °C/min, heating from −50 °C to 200 °C at 10 °C/min.

### 2.4. IR Measurements

The infrared spectra were recorded on the Nicolet iS10 FT-IR spectrometer (Thermo Scientific, Waltham, MA, USA) using an ATR sampling module, on diamond crystal, in the spectral range from 4000 to 650 cm^−1^ and a spectral resolution of 4 cm^−1^. For one spectrum, 200 scans were recorded. 

### 2.5. Mechanical Parameters of the Printlets and Apparent Density of Printlets

The hardness of the printlets was evaluated according to European Pharmacopoeia 2.9.8 Resistance to crushing General Monograph. The test was carried out on 10 tablets in hardness tester YD-3 (Hinotek Group Ltd., Ningbo, China).

The apparent density of the printlets was calculated by dividing the weight of the printlets of a particular formulation and the volume of the printout. For this purpose, 10 printlets of each formulation were weighed accurately, the apparent density was calculated. Due to the printing precision, the volume of the printlets was treated as constant (1.152 cm^3^).

### 2.6. Drug Release Studies and Buoyancy Observation

Met release studies were performed using USP Apparatus 3 and Apparatus 4, according to The European Pharmacopoeia (Ph. Eur.).

The USP Apparatus 3 dissolution system consisted of a BIO-DIS Reciprocating Cylinder Apparatus G7970-64011 in closed on-line configuration with Agilent 89092-60002 Multichannel Pump paired with an Agilent Cary 8454 UV-Vis spectrophotometer and controlled by UV-Visible GLP Chem Station Software v.B.05.04 (all from Agilent Technologies Inc., Penang, Malaysia). Seven vessels filled with 250 mL of 0.01 M HCl pH 2.0 dissolution medium were placed at 37 ± 0.5 °C. The apparatus was set at 15 dips per minute (DPM). 

The release of Met in Apparatus 4 was carried out in a closed-loop, semi-automated flow-through cell dissolution system (SOTAX AG, Aesch, Switzerland). The dissolution system consisted of a SOTAX CE 7 smart unit with a set of seven 22.6 mm internal diameter cells, SOTAX CP 7-35 piston pump, and single-beam Agilent Cary 8454 UV-Vis spectrophotometer controlled by WinSOTAX Plus Dissolution Software v.2.60.4.1. As a dissolution medium, 0.01 M HCl, pH 2.0 in a volume of 250 mL was used. 

## 3. Results and Discussion

Formulation A (80/20/0/100) was a basic composition. This formulation was modified to analyze the influence of changes of several factors on the properties of the FDDS. The possibility of introducing an osmotic substance into the matrix was tested on Formulation B (80/18/2/100). In the case of Formulation C (80/20/0/150), the influence of laser speed was tested. Formulation D (90/10/0/75) was prepared to test the possibility of decreasing PA12 content. In this case, the laser speed was reduced to obtain the acceptable printout.

### 3.1. The Characteristics of the Printlets

The technical drawing of the model and photo of the printlet manufactured by SLS are presented in Figure 1a,b, respectively. All formulations obtained with this technique were regular shapes without damage or chipping. The surface of the printlets was rough, which suggested that the final formulation should be coated or encapsulated to enable their swallowing. The mean weight of the tablets was 800 mg (n = 10), and the weight distribution was in the range ±5% (760–840 mg). The hardness of the printlets was similar for all developed formulations. It was in the range 37–49 N. The mean hardness of the printlets was: Formulation A (80/20/0/100) 45 N, Formulation B (80/18/2/100) 40 N, Formulation C (80/20/0/150) 43 N and Formulation D (90/10/0/75) 44 N.

**Figure 2:**
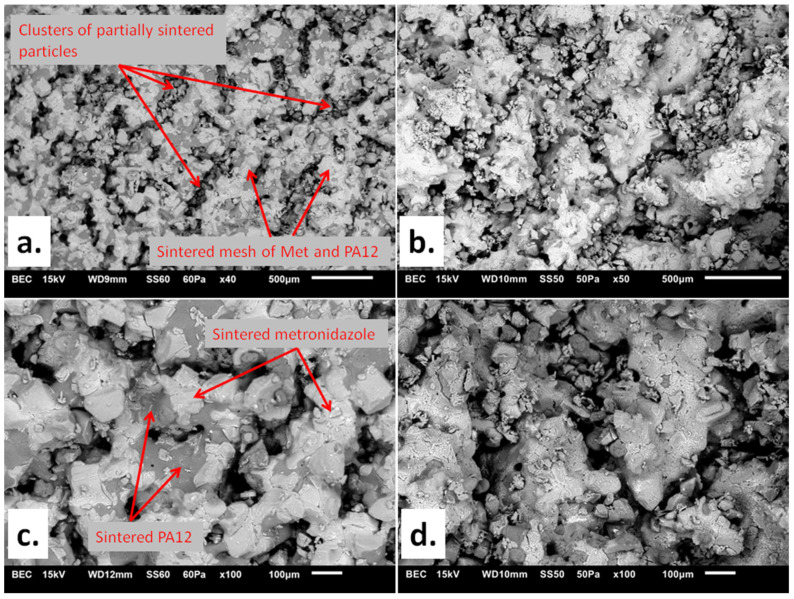
SEM images of (**a**) Formulation A (80/20/0/100) at 40× magnification; (**b**) Formulation D (90/10/0/75) at 50× magnification; (**c**) Formulation A (80/20/0/100) at 100× magnification; (**d**) Formulation D (90/10/0/75) at 100× magnification. The markings are commented on in the text.

Figure 2 shows SEM images of Formulations A (80/20/0/100) and D (90/10/0/75) at two different magnifications. The printlets had a composite structure with zones where the material was only slightly sintered. The EDS applied to analyze the various spots in the images showed differences in carbon content, suggesting that they were built with different materials (Supplementary Materials, Figures S1–S7). The darker areas in the images corresponded to PA12, and the lighter ones belonged to crystalline clusters of Met.

**Figure 3:**
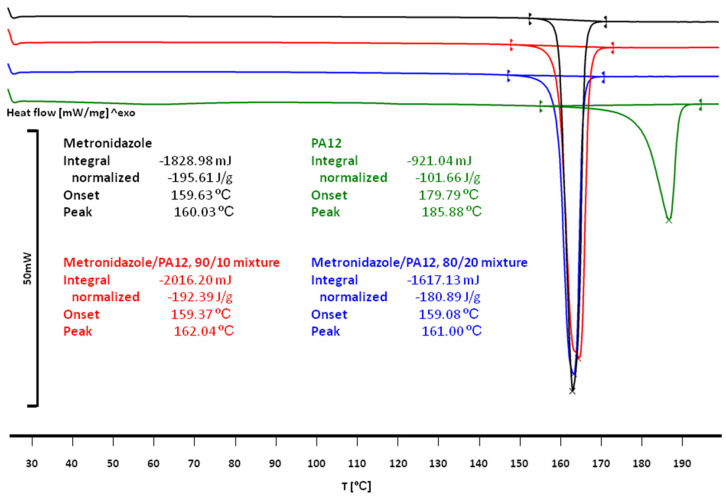
DSC curves of pure Met, PA12 and physical mixtures Met/PA12 (90/10 and 80/20).

The thermal studies of raw materials applied for printlet preparation (Figure 3) showed that the melting point for Met was about 160 °C, while the PA12 had a melting point of about 186 °C. The PA12 particles absorbed the laser energy more effectively as they contained dye, and the sintering process could be described in two steps. (1) Since the glass transition temperature (T_g_) of PA12 is about 50 °C [42], it is believed that the laser energy kept the amorphous regions of PA12 in a viscous state. Sintered PA12 formed the mesh (continuous structure). (2) Thermal energy was also transferred to Met crystals—smaller ones (microscopic observations confirmed their presence) could be sintered together with adjacent Met crystals and PA12. Some non-sintered grains were visible in the pore space of Formulations A and D, suggesting that energy transfer was inefficient. 

Formulations A (80/20/0/100) and D (90/10/0/75) required independent selection of sintering parameters due to different dye content. In the case of Formulation D (90/10/0/75), more energy was needed per time unit to obtain printlets with an appropriately-formed internal structure (Figure 2c,d). It required a slower laser speed (75 mm/s) during printing.

### 3.2. The Effect of Sintering on the Phase Transitions of Printlet Components 

DSC and IR techniques were used to evaluate the phase transitions of nylon and Met during sintering. The PA12 exists in four crystal forms: α, α′, γ and γ′ [42,43,44,45]. Both γ and γ′ forms exhibit a single melting peak at 177.5 °C and 176 °C, respectively. In the case of the α form, two melting effects at 168.5 °C and 177 °C are observed in a DSC curve. Studies performed by Dadbakhsh et al. [44] have shown that the crystal structure of the powder changes from α to γ-form after SLS melting. The change is associated with a drastic drop in crystallinity, as observed using wide-angle X-ray scattering. However, Martynková et al. [45] have proved that laser sintering decreases the lamellar crystallite size of PA12, calculated from powder diffractograms because the partial phase transformation from γ to α may reduce lamellar crystallite size due to disruptions caused in the γ lamellar stacking continuum. 

The thermal studies of the components of the printlets were in agreement with literature data. The DSC curves of Met and PA12 were characterized by single endothermic peaks (Figure 3). Met melted at 159.63 °C (ΔH = −195.61 J/g), which was in agreement with the data published by Agafonova et al. [46]. The PA12 melted at 179.79 °C (ΔH = −101.66 J/g), which agreed with the melting characteristic of γ-form [42,44]. 

In both Met/PA12 mixtures and Formulation A (80/20/0/100) and Formulation D (90/10/0/75), the melting effect was observed only for Met. The melting temperature and enthalpy moved to lower values for Met/PA12 mixtures, i.e., 159.37 °C, −192.39 J/g for the 90/20 mixture and 159.08 °C, −180.89 J/g for 80/20 mixture.

**Figure 4:**
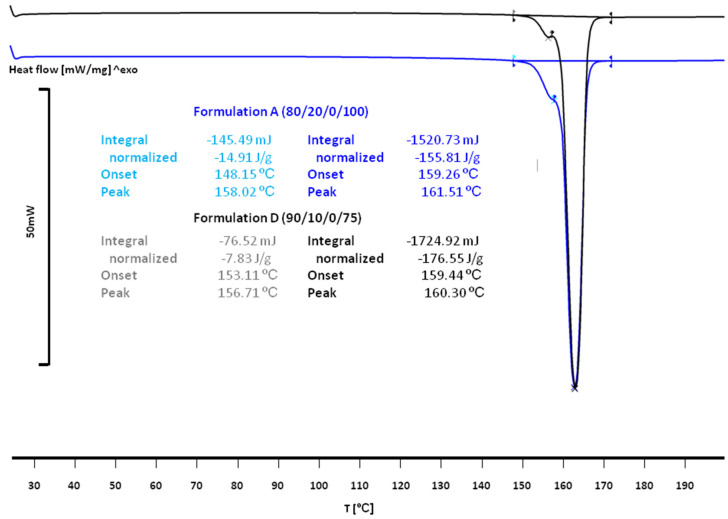
DSC curves of printlets from Formulation A (80/20/0/100) and Formulation D (90/10/0/75).

DSC curves of printlets are presented in Figure 4. They differed from the DSC curves of the mixtures by a small additional endothermic effect on the left arm of the main melting effect of Met. The melting temperatures and enthalpies for Formulations A (80/20/0/100) and D (90/10/0/75) were different due to different Met/PA12 ratios (Figure 3). Thermal parameters for Formulation A (80/20/0/100) printlets were 148 °C, −15 J/g for the first effect and 159 °C, −156 J/g for the second effect. For Formulation D, the first effect was observed at 153 °C, with enthalpy −8 J/g, and the second effect was detected at 159 °C, −177 J/g. It was supposed that the first melting effect originated from phase transitions between γ and α form of PA12, with accompanying changes in crystallinity [45]. 

DSC thermograms of Met/PA12 mixtures confirmed the presence of Met. An additional, small endothermic effect was observed in DSC curves of printlets apart from the pure Met melting effect. It was supposed that this small effect originated from polymorphic transitions of PA12. 

**Figure 5:**
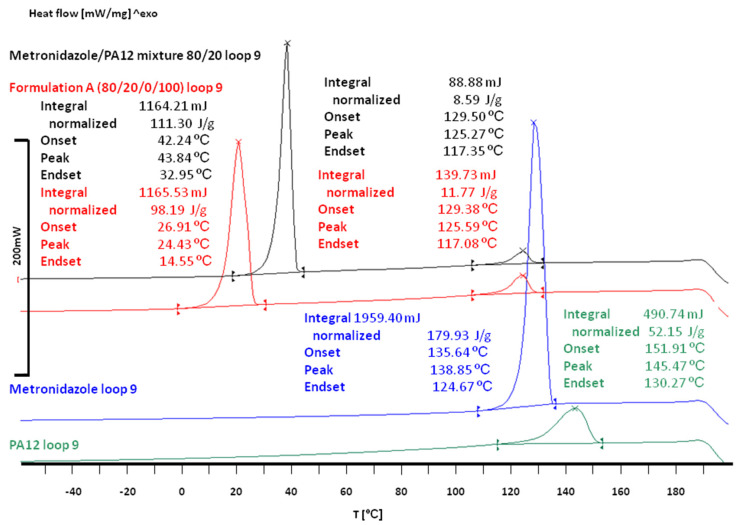
DSC curves from the thermal loop of Met (blue), PA12 (green), mixture (black) and Formulation A printlet (red); the 3rd segment-cooling from 200 °C to −60 °C.

The additional temperature program, consisting of 5 segments, was used to detect phase transitions in the tested samples (a complete set of raw DSC curves obtained from this thermal loop is presented in the Supplementary Materials, Figures S8–S12). The third segment (cooling from 200 °C to −60 °C) of the loop (Figure 5) contains the most interesting results. In curves obtained for powder mixtures as well as for printlets, two crystallization effects were observed at about 129 °C (the same for the mixture and the sintered sample), 42 °C (for the mixture) and 27 °C (for the sintered sample). From the comparison of crystallization enthalpies of PA12 and Met with those obtained from the mixture and the sintered sample, it could be assumed that the effect at 129 °C reflected crystallization of PA12, the effect at 42 °C and 27 °C originated from the crystallization of Met. It is worth indicating a significant difference between the crystallization temperature of Met alone and Met in the mixture or the sintered sample. Moreover, the crystallization temperature of Met in the sintered sample was downshifted by about 15 °C in comparison to the mixture, which can prove additional effects in the printlet. 

In ATR-IR spectra, Met was easily identified by the intensive bands at 3209 cm^−1^ (from O-H stretching vibrations), 3100 cm^−1^ (from C-H stretching vibrations), 1534 cm^−1^ (from -NO_2_ asymmetric stretching vibrations), 1472 cm^−1^ and 1427 cm^−1^ (from C-H deformation vibrations), 1367 cm^−1^ (from -NO_2_ symmetric stretching vibrations), 1186 cm^−1^ (from C-O stretching vibrations) and 1073 cm^−1^ (from the ring breathing mode) [47]. 

PA12 forms were well distinguished utilizing IR spectroscopy. The most significant differences between forms were for the amide II band. The origin of this band in secondary amides was due to a mixed vibration. The mixing was between the N-H in-plane bending and the C–N stretching vibration. This band appeared at 1561 cm^−1^ in γ-PA12, 1557 cm^−1^ in γ′-PA12, and in 1545 cm^−1^ in α-PA12 [43].

**Figure 6:**
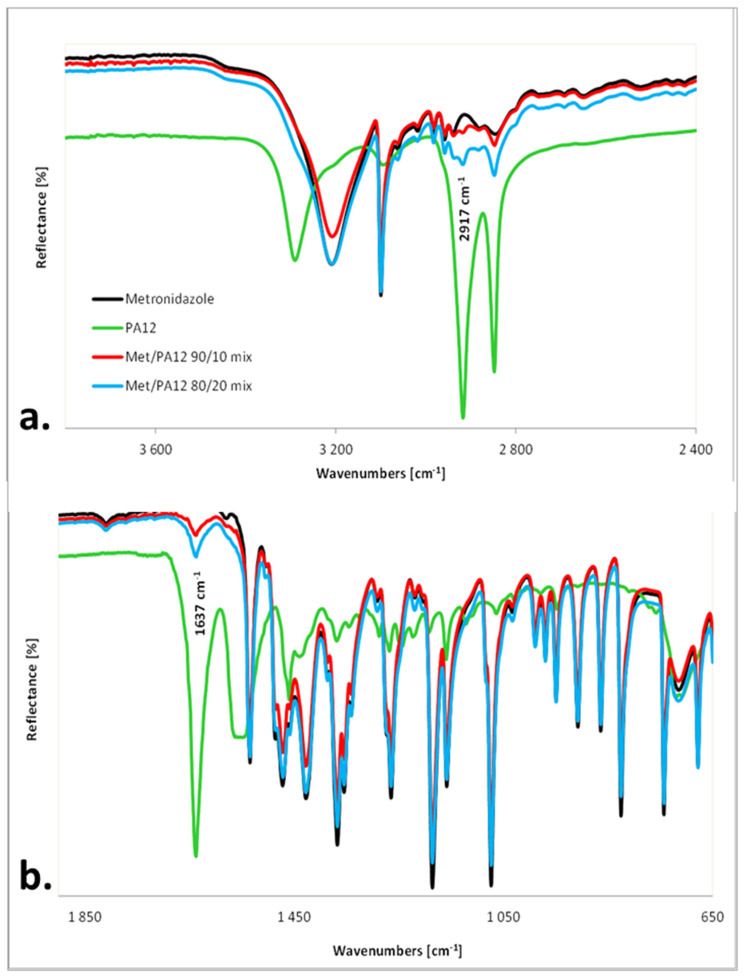
IR-ATR spectra of the pure components and physical mixtures of powders (**a**) in the range of 3700–2400 cm^−1^; (**b**) in the range of 1850–650 cm^−1^.

Nylon PA12 was characterized by the intensive bands at 3290 cm^−1^ (from N-H stretching vibrations), 2917 cm^−1^ and 2848 cm^−1^ (from C-H asymmetric and symmetric stretching vibrations), 1637 cm^−1^ (from C=O stretching vibrations, amide I Band), and broadband from 1569 to 1540 cm^−1^ (from the N-H in-plane bending and the C–N stretching vibration, amide II Band) indicating the presence of a mixture of PA12 forms [42,44]. In the spectra of both mixtures, mainly Met bands were observed as well as two characteristic bands at 2971 cm^−1^ (Figure 6a) and 1637 cm^−1^ (Figure 6b) from PA12. 

**Figure 7:**
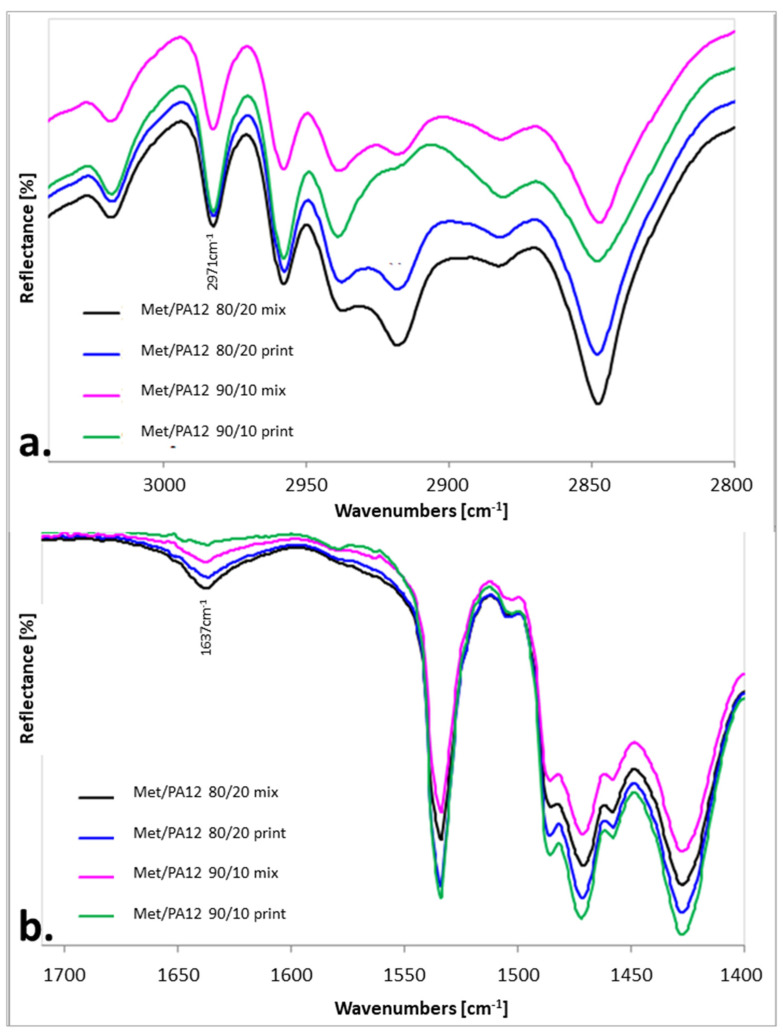
IR-ATR spectra of the printlets and corresponding physical mixtures (**a**) in the range of 3040–2800 cm^−1^; (**b**) in the range of 1710–1400 cm^−1^.

The IR spectroscopy confirmed the presence of Met and PA12 in mixtures and printlets. However, due to a small percentage of PA12, only two weak bands were visible in the IR spectra of mixtures and tablets (Figure 7). As a result, there were no visible differences and band shifts between the IR spectra of the mixtures and the printlets. It indicated the lack of interaction between Met and PA12 in the printlet.

The results presented in this section, together with literature data, gave the strong premises that the composite material was obtained, i.e., elastic insoluble PA12 mesh filled with brittle crystalline Met.

### 3.3. Drug Release Studies and Floating Properties

The drug release studies were carried out using two different pharmacopeial methods: reciprocating cylinder (Apparatus 3 Ph. Eur./USP) and flow-through cell (Apparatus 4 Ph. Eur./USP). Combining both methods allowed for a comprehensive assessment of the printlets’ properties. In the reciprocating cylinder apparatus (Apparatus 3), the repeating movements of the cylinders during the dips exerted additional effects on the printlets to simulate the mechanical stress in the stomach [48,49], but the flotation of the printlets was not possible to observe. The flow-through cell apparatus was applied to assess the influence of the formulation on drug dissolution in mild conditions, as well as the floating behavior of the printlets. 

**Figure 8:**
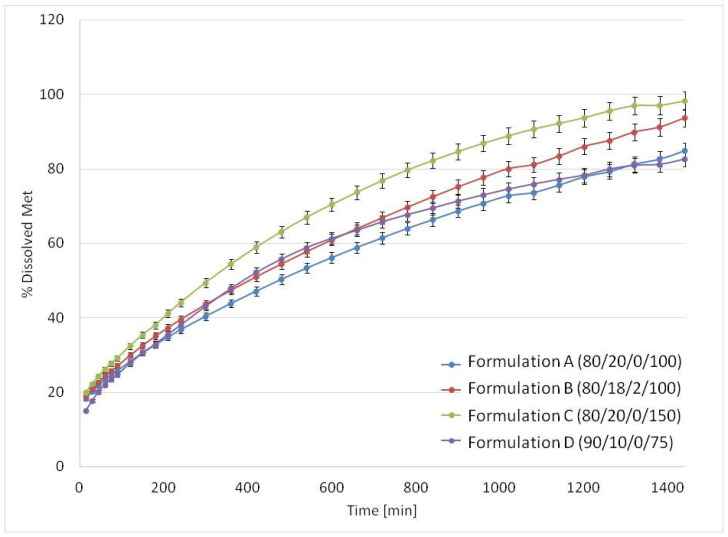
The mean release profiles (*n* = 3) of Met from printlets in the USP4 apparatus.

Drug dissolution in USP apparatus 4 was relatively slow. The release profiles of Met are presented in Figure 8. The time required to release 80% of drug substance was longer than 12 h, i.e., 13 h for Formulation C (80/20/0/150), 17 h for Formulation B (80/18/2/100) and 21 h for Formulations A (80/20/0/100) and D (90/10/0/75).

**Figure 9:**
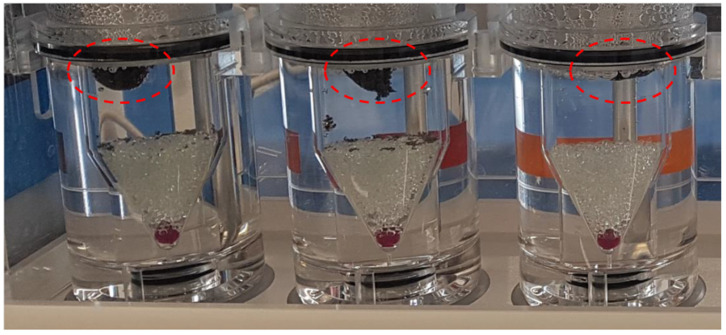
Flotation of Formulation C (80/20/0/150) in USP4 apparatus after 10 h of the experiment.

For all formulations, the mean apparent density was lower than 0.8 g/cm^3^ (Table 2). The porous structure of the printlets ensured their immediate flotation after the filling of flow-through cells. Due to the low content of insoluble PA12 the matrices were gradually fragmented and, in most cases, the printlets sunk within the first hour of the experiment. However, in the case of Formulation C (80/20/0/150), which had the lowest apparent density, flotation of the matrices was observed for 10 h (Figure 9). The observations confirmed that the porous structure of the printlets allowed their flotation. This feature could be optimized in further studies. 

**Figure 10:**
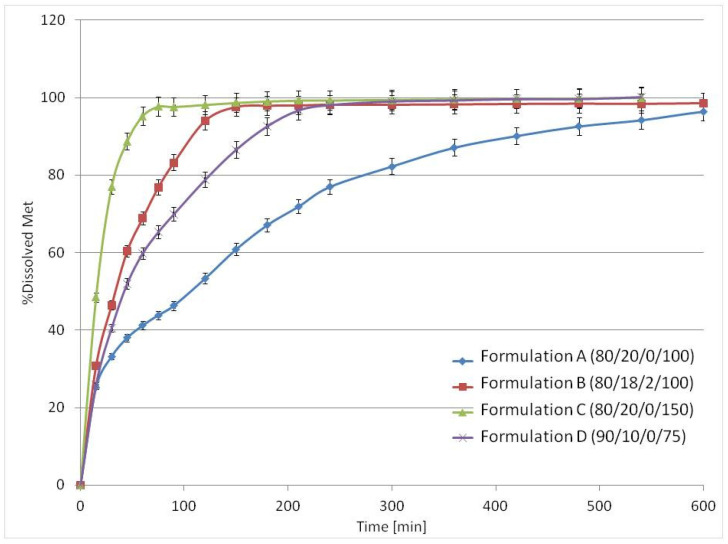
The mean release profiles (*n* = 3) of Met from 3D printlets in the USP3 apparatus.

Met release in USP apparatus 3 was substantially faster than in USP4 for all formulations due to different hydrodynamic conditions (Figure 10). Dissolution from Formulation A (80/20/0/100) was biphasic. Up to 4 h it was almost linear, and Formulation A released 80% of API at 5 h with the full drug release longer than 10 h. Regarding Formulation C (80/20/0/150), the standard composition containing 80% *v/v* of Met with higher laser speed (150 mm/s) resulted in a much faster Met release—about 80% of the dose was released during the first 30 min, and complete release occurred at 2.5 h. The interesting modification of the 80/20 formulation was replacing 2% PA12 with the same volume of sodium chloride as the osmotic agent in Formulation B (80/18/2/100). The presence of sodium chloride in the matrix modified Met release from the printlets. They released about 80% of the API within 1.5 h, and the total Met release was observed at 2.5 h. While the melting point of NaCl is high (801 °C) compared with other components, the addition of NaCl is unlikely to affect the sintering process. The explanation of faster release could be an increase in the osmotic pressure of the solution present in the porous printlet structure and, consequently, more effective water penetration into the matrix. For formulation containing only 10% *v/v* of PA12, Formulation D (90/10/0/75), almost 80% of Met was released during the first 2 h. However, in this case, faster release than for 80/20 formulation was probably caused by the weaker composite structure due to the lower PA12 content. 

### 3.4. General Discussion

The current study combined the very early concepts in DDS manufacturing by SLS, where a water-insoluble (nylon or polycaprolactone) matrix has been used [50,51,52,53,54,55], and the idea of manufacturing highly drug-loaded printlets with SLS, which is presented on paracetamol printlets by Kulinowski et al. [36]. For example, fine nylon powder has been sintered into matrices, which have been soaked with dye (methylene blue) as a drug model [51]. In another study, biodegradable polycaprolactone matrices loaded with progesterone feature zero-order kinetics [54,55]. The drug release from such DDS has been slow, with full drug release from several to dozens of days. Such structures can be useful when a long release time is required (e.g., implants) and are inadequate as oral drug delivery systems. On the other hand, obtaining a product with good mechanical properties by sintering pure, stained API is a very demanding process [36]. Putting a high content of API into insoluble elastic thread with low polymer content resulted in a composite material of improved mechanical properties and printability. 

The potential of high drug loading, i.e., 80% *w/w* and more, which could be obtained without sacrificing printability and mechanical properties, cannot be overestimated. The work by Thakkar et al. shows the mechanical properties of the printlets manufactured using pharmaceutical grade polymer and containing 5–10% *w/w* of API [56]. The best tensile strength obtained in their study is comparable with composite Met/PA12 formulations containing up to 90% *v/v* (more than 90% *w/w*) of the brittle API.

Nylons are good candidates as potential pharmaceutical excipients due to their biocompatibility (see the extensive review article by Shakiba et al. [38]), printability and good mechanical properties. At this stage of DDS development, using technical grade (carbon-stained nylon) had no negative impact on the presentation of the general idea of the study and even on further developments. Such an approach was justified as no pharmaceutical-grade nylons are optimized for SLS printing.

## 4. Conclusions

Classical oral dosage forms achieve mechanical strength due to powder compression. As a result, pharmaceutical oral dosage forms excipients are designed primarily for powder compression. Using pharmaceutical-grade excipients frequently leads to poor mechanical characteristics of the printlets manufactured with SLS technology. Therefore, there is an unmet need to seek a new set of dedicated materials matching 3D printing technologies. In the presented study, the composite material was obtained consisting of elastic insoluble PA12 mesh filled with high content of brittle (crystalline) Met using SLS. As mesh consisted of 10 or 20% of the printlet, a high therapeutic API dose could be incorporated, i.e., ca. 600 mg of Met. Despite the high content of brittle drug substance, good mechanical properties were obtained. Together with the potential gastroretentive properties of the printlets, obtained drug delivery systems have the potential for *H. pylori* treatment. Differences in thermal characteristics between powder mixtures and printlets were identified by DSC experiments. It was shown that the drug-excipients mixture could generate complicated thermal patterns in terms of DSC curves which should be accounted for when selecting printing parameters. An additional potential degree of freedom for shaping/tuning drug release profiles was introduced. The addition of a low amount of osmotic agents, in our case 2% *v/v* of NaCl, could influence drug dissolution from composite PA12/Met printlets. The developed ideas can be used to design other printlet formulations.

The properties of standard pharmaceutical excipients applied for tableting result from decades of experiments and experience gathered during the optimization of the manufacturing processes of drug delivery systems. The study shows the need to define the requirements for excipients dedicated to 3D printing and to search for appropriate materials for this purpose.

## Figures and Tables

**Table 1:** Compositions expressed as volumetric (*_v/v_*) and weight (*_w/w_*) percent of components in printlets formulations and laser speed parameters.

	Metronidazole		PA12		Sodium Chloride		Laser Speed (mm/s)
	(% *_v/v_*)	(% *_w/w_*)	(% *_v/v_*)	(% *_w/w_*)	(% *_v/v_*)	(% *_w/w_*)	
Formulation A (80/20/0/100)	80	84.1	20	15.9	0	0	100
Formulation B (80/18/2/100)	80	81.7	18	13.9	2	4.4	100
Formulation C (80/20/0/150)	80	84.1	20	15.9	0	0	150
Formulation D (90/10/0/75)	90	92.2	10	7.8	0	0	75

**Table 2:** Mean apparent density of formulations obtained by SLS (*n* = 10) and results of buoyancy observations in Apparatus 4.

	Formulation A (80/20/0/100)	Formulation B (80/18/2/100)	Formulation C (80/20/0/150)	Formulation D (90/10/0/75)
Mean apparent density (g/cm^3^)	0.76	0.73	0.62	0.82
SD (*n* = 10)	0.02	0.02	0.01	0.02
Buoyancy lag time (min)	0	0	10	0
Total floating time (min)	30	60	600	100

## Data Availability

The processed data required to reproduce these findings is available from the authors upon reasonable request.

## Supplementary Materials

The following supporting information can be downloaded at: https://www.mdpi.com/article/10.3390/ma15062142/s1, Figure S1: SEM with identified spots, where EDS spectra were recorded, Figure S2: The EDS spectrum recorded at spot 4, Figure S3: The EDS spectrum recorded at spot 5, Figure S4: The EDS spectrum recorded at spot 6, Figure S5: The EDS spectrum recorded at spot 7, Figure S6: The EDS spectrum recorded at spot 8, Figure S7: The EDS spectrum recorded at spot 9, Figure S8: DSC curves from thermal loop of Metronidazole (blue), PA12 (green), mixture (black) and printed tablet (red), Figure S9: DSC curves from thermal loop of Metronidazole segment 1 and 4-melting, Figure S10: DSC curves from thermal loop of PA12, segment 1 and 4-melting, Figure S11: DSC curves from thermal loop of mixture, segment 1 and 4-melting, Figure S12: DSC curves from thermal loop of printed tablets, segment 1 and 4-melting.
