# Supplementary material for: Development of Composite, Reinforced, Highly Drug-Loaded Pharmaceutical Printlets Manufactured by Selective Laser Sintering—In Search of Relevant Excipients for Pharmaceutical 3D Printing
Source: Materials (Basel). 2022 Mar 14;15(6):2142. doi: 10.3390/ma15062142 (PMC8950795; doi:10.3390/ma15062142)
Supplement: Supplementary file 1 [file materials-15-02142-s001.zip › materials-1603134-supplementary.pdf]

materials

Supplementary Materials

Development of Composite, Reinforced, Highly Drug-Loaded Pharmaceutical Printlets Manufactured by Selective Laser Sintering—In Search of Relevant Excipients for Pharmaceutical 3D Printing

Piotr Kulinowski [1], Piotr Malczewski [1], Marta Łaszcz [2], Ewelina Baran [1], Bartłomiej Milanowski [3,4], Mateusz Kuprianowicz [4] and Przemysław Dorożyński [5,6,*]

[1] Institute of Technology, Pedagogical University of Cracow, Podchorążych 2, 30-084 Cracow, Poland; piotr.kulinowski@up.krakow.pl (P.K.); pmalczewski@gmail.com (P.M.); ewelina.baran@up.krakow.pl (E.B.)
[2] Department of Falsified Medicines and Medical Devices, National Medicines Institute, Chełmska 30/34, 00-725 Warsaw, Poland; m.laszcz@nil.gov.pl
[3] Chair and Department of Pharmaceutical Technology, Poznan University of Medical Sciences, ul. Grunwaldzka 6, 60-780 Poznan, Poland; b.milanowski@rcz-zbaszyn.pl
[4] GENERICA Pharmaceutical Lab, Regionalne Centrum Zdrowia Sp. z o.o., Na Kępie 3, 64-360 Zbąszyń, Poland; m.kuprianowicz@rcz-zbaszyn.pl
[5] Department of Drug Technology and Pharmaceutical Biotechnology, Medical University of Warsaw, Banacha 1, 02-097 Warsaw, Poland
[6] Department of Spectroscopic Methods, National Medicines Institute, Chełmska 30/34, 00-725 Warsaw, Poland
* Correspondence: mfdorozy@cyf-kr.edu.pl

Citation: Kulinowski, P.; Malczewski, P.; Łaszcz, M.; Baran, E.; Milanowski, B.; Kuprianowicz, M.; Dorożyński, P. Development of Composite, Reinforced, Highly Drug-Loaded Pharmaceutical Printlets Manufactured by Selective Laser Sintering—In Search for Relevant Excipients for Pharmaceutical 3D Printing. *Materials* **2022**, *15*, 2142. https://doi.org/10.3390/ma15062142

Academic Editor: Ana Paula Piedade

Received: 2 February 2022
Accepted: 9 March 2022
Published: 14 March 2022

Publisher's Note: MDPI stays neutral with regard to jurisdictional claims in published maps and institutional affiliations.

Copyright: © 2022 by the authors. Licensee MDPI, Basel, Switzerland. This article is an open access article distributed under the terms and conditions of the Creative Commons Attribution (CC BY) license (http://creativecommons.org/licenses/by/4.0/).

Energy Dispersion Spectroscopy

The chemical analysis was performed with energy dispersion spectroscopy (EDS) using the X-Max detector, equipped with Aztec 2.1 software (Oxford Instruments, Oxford, UK).

In Figures S1-S7, SEM with identified spots, where EDS spectra were recorded, and corresponding spectra are presented.

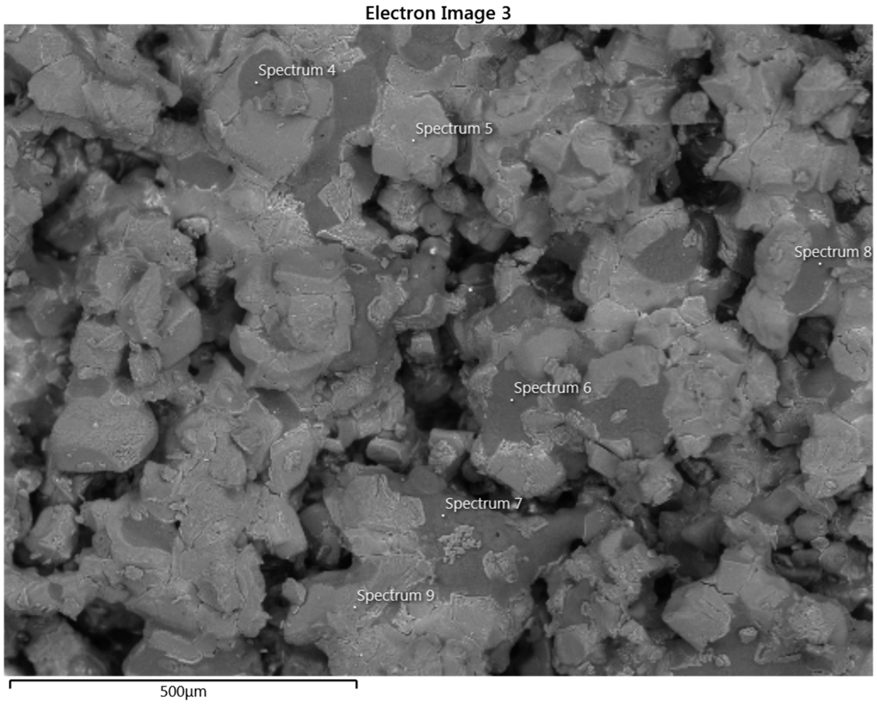

Figure S1. SEM with identified spots, where EDS spectra were recorded.

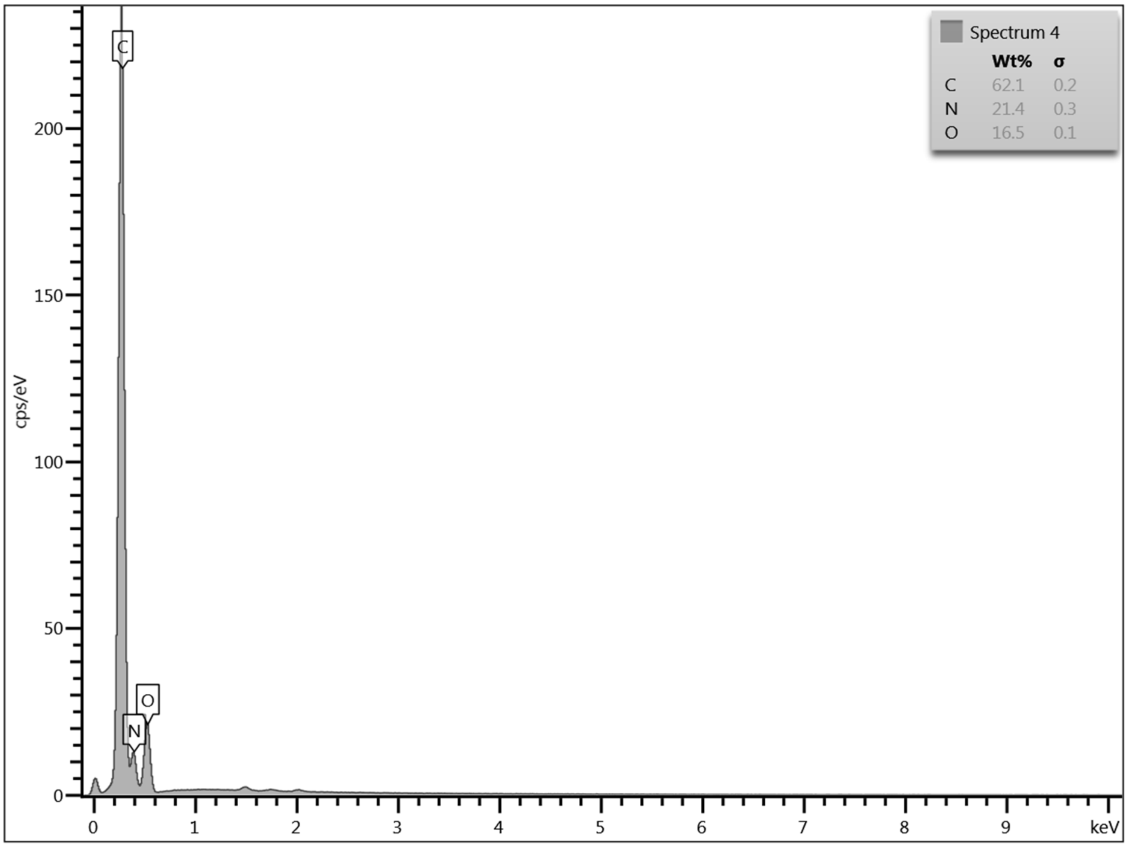

Figure S2. The EDS spectrum recorded at spot 4.

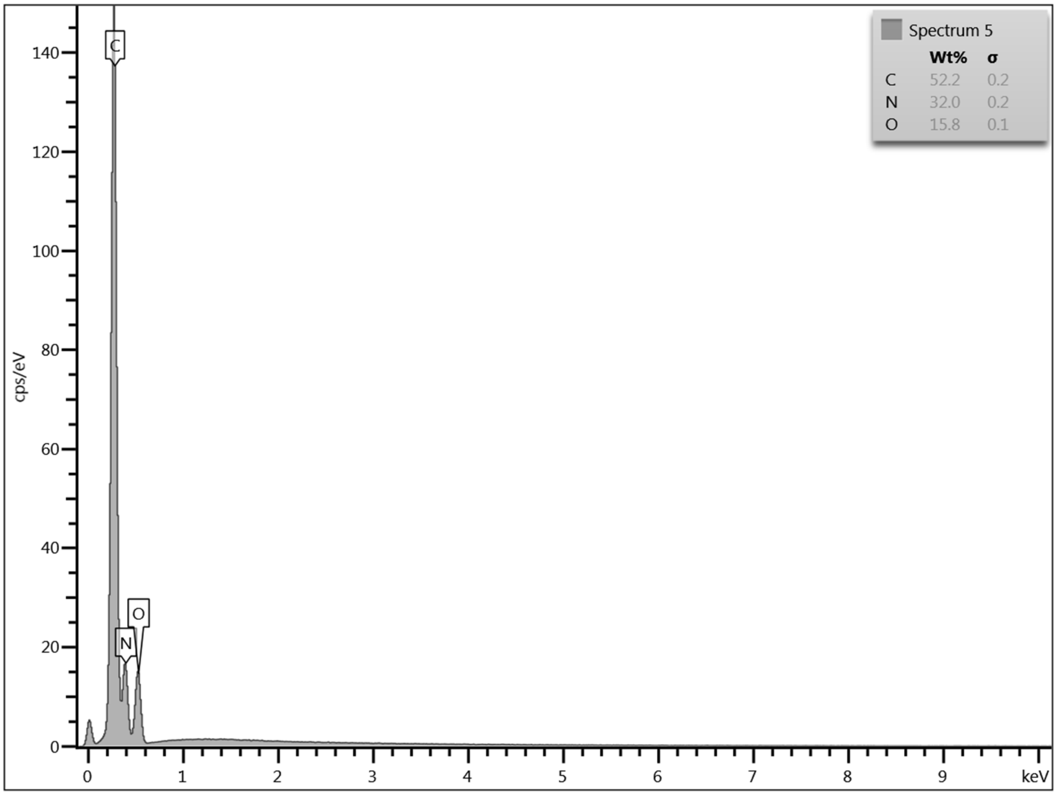

Figure S3. The EDS spectrum recorded at spot 5.

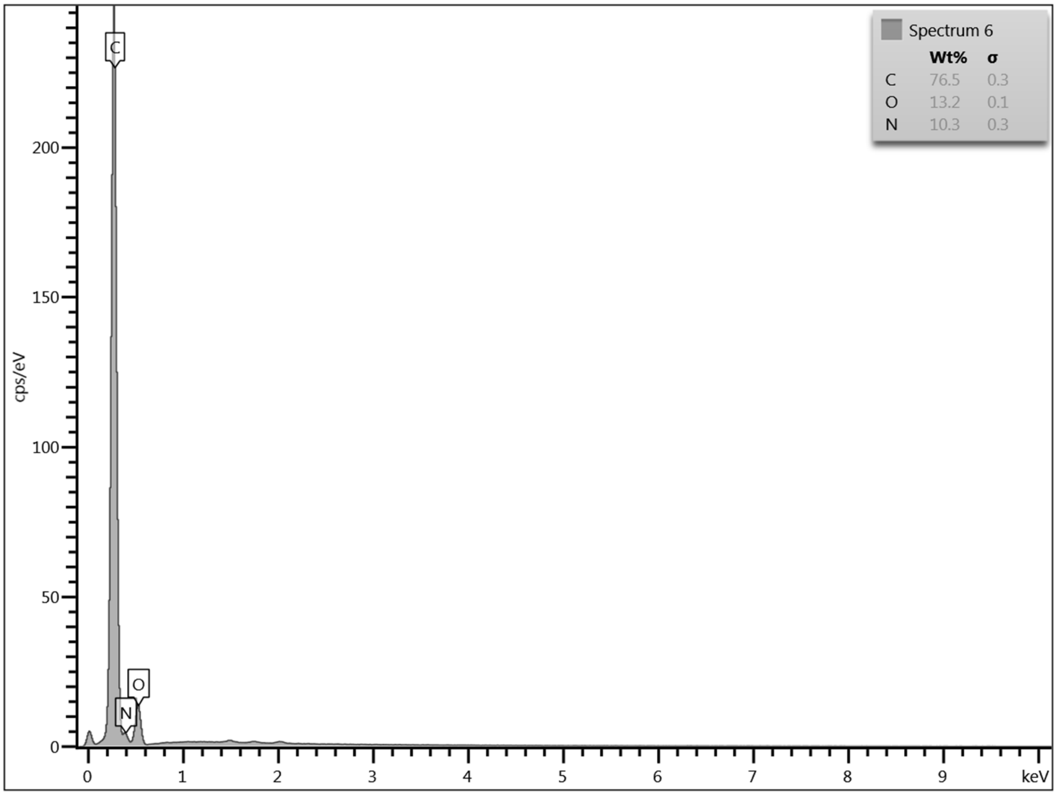

Figure S4. The EDS spectrum recorded at spot 6.

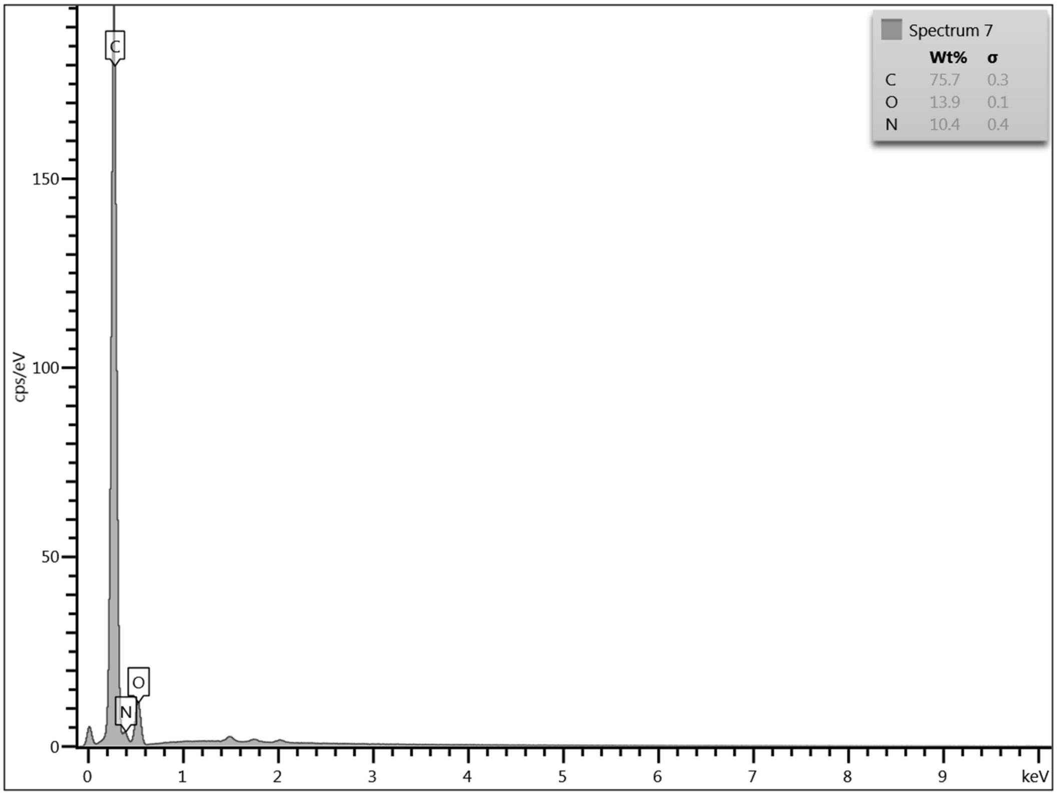

Figure S5. The EDS spectrum recorded at spot 7.

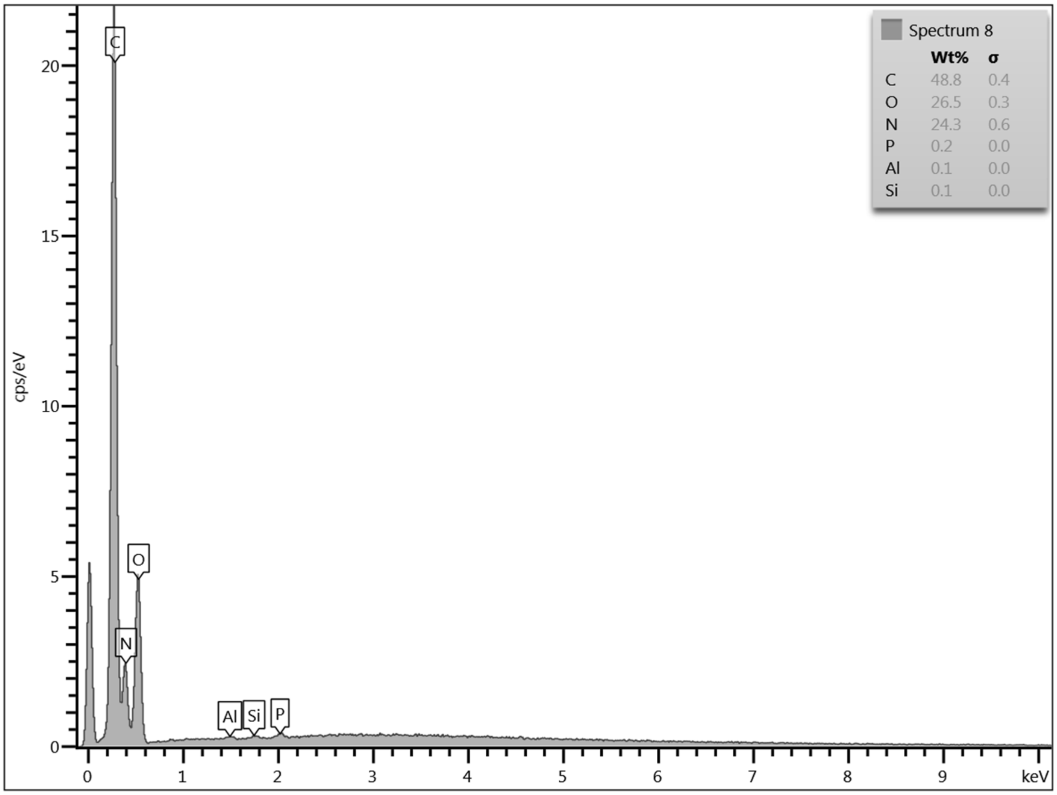

Figure S6. The EDS spectrum recorded at spot 8.

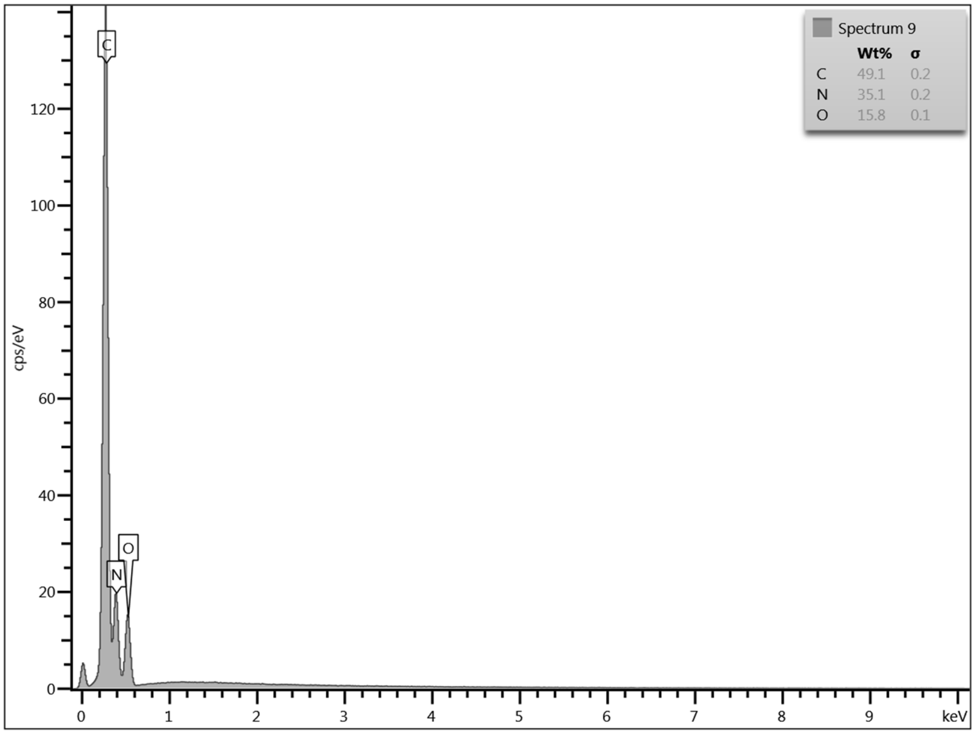

Figure S7. The EDS spectrum recorded at spot 9.

DSC

Thermal loop experiments were carried out to detect phase transitions in the tested samples. Figure S8 shows DSC curves obtained during these experiments and the temperature program.

Figures S8–S12 show the DSC curves of the 1st and 4th segment of the loop for Metronidazole, PA12, the mixture and the sintered sample, respectively.

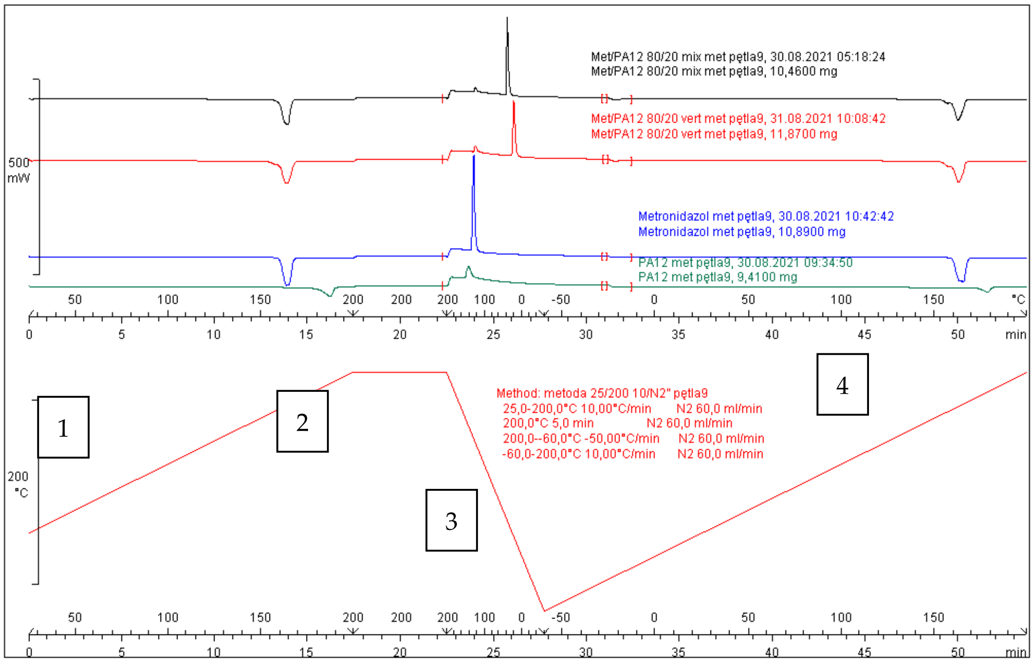

Figure S8. DSC curves from thermal loop of Metronidazole (blue), PA12 (green), mixture (black) and printed tablet (red).

The temperature program:

1. 25-200 °C, 10 °C/min
2. 200 °C 5 min
3. 200– 60 °C, 50 °C/min
4. −50 °C –200 °C, 10 °C/min

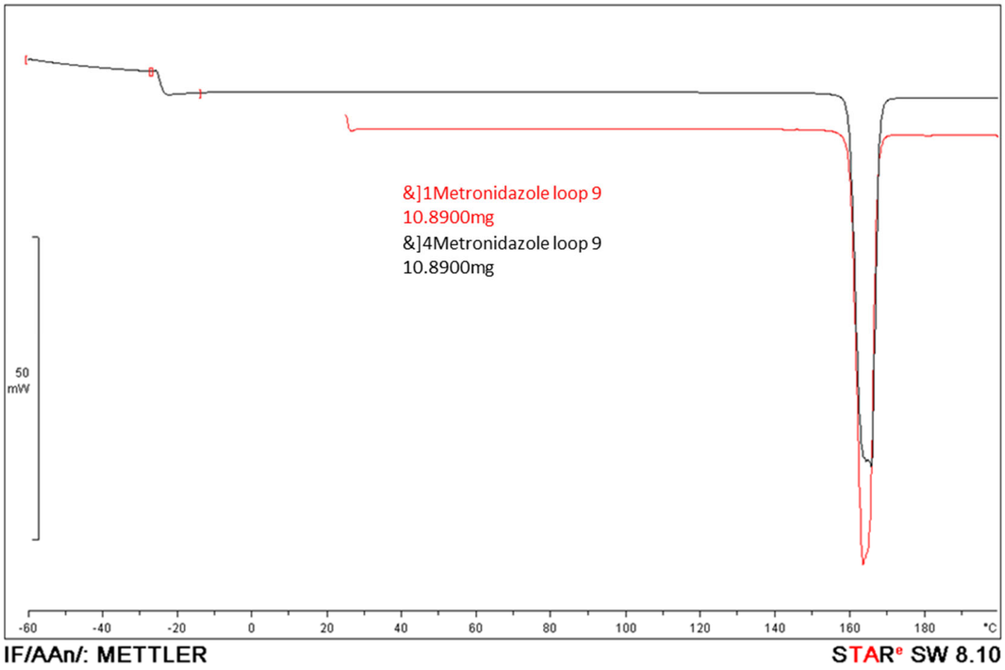

Figure S9. DSC curves from thermal loop of Metronidazole segment 1 and 4 – melting.

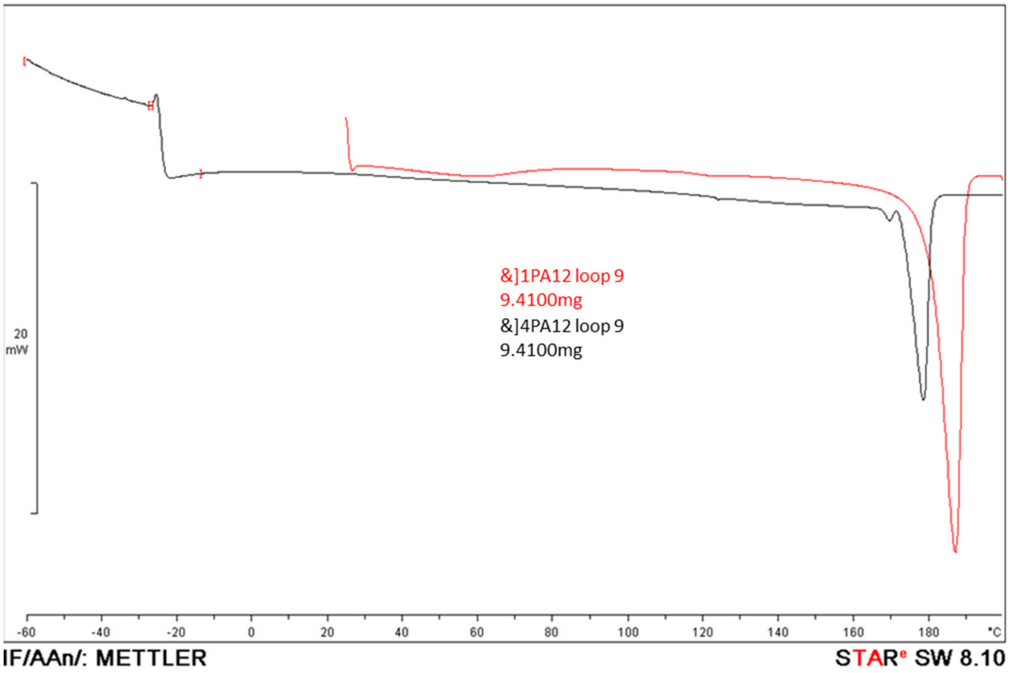

Figure S10. DSC curves from thermal loop of PA12, segment 1 and 4 – melting.

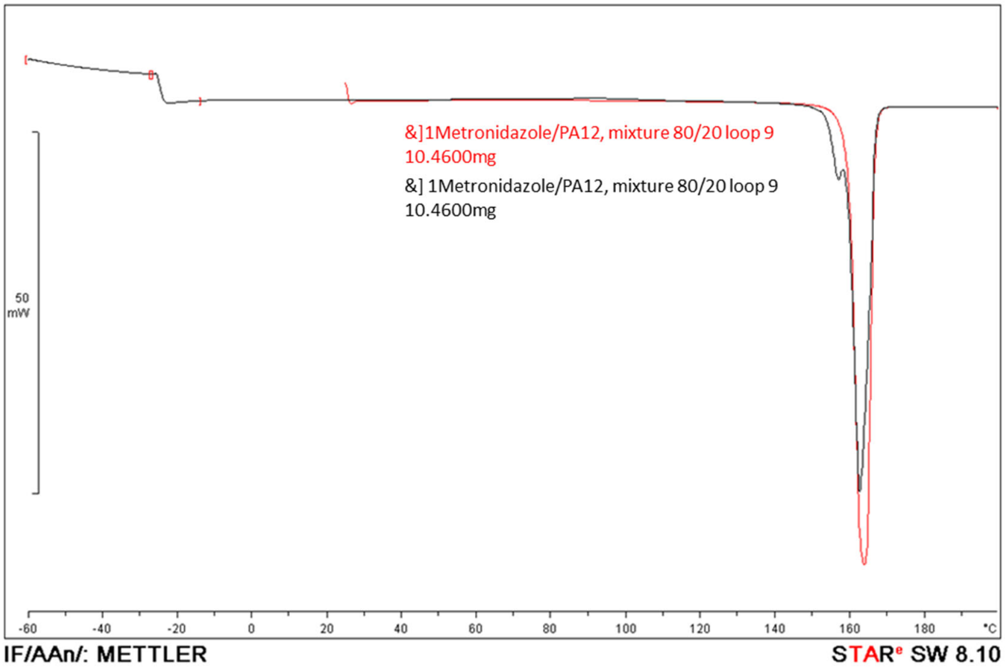

Figure S11. DSC curves from thermal loop of mixture, segment 1 and 4 – melting.

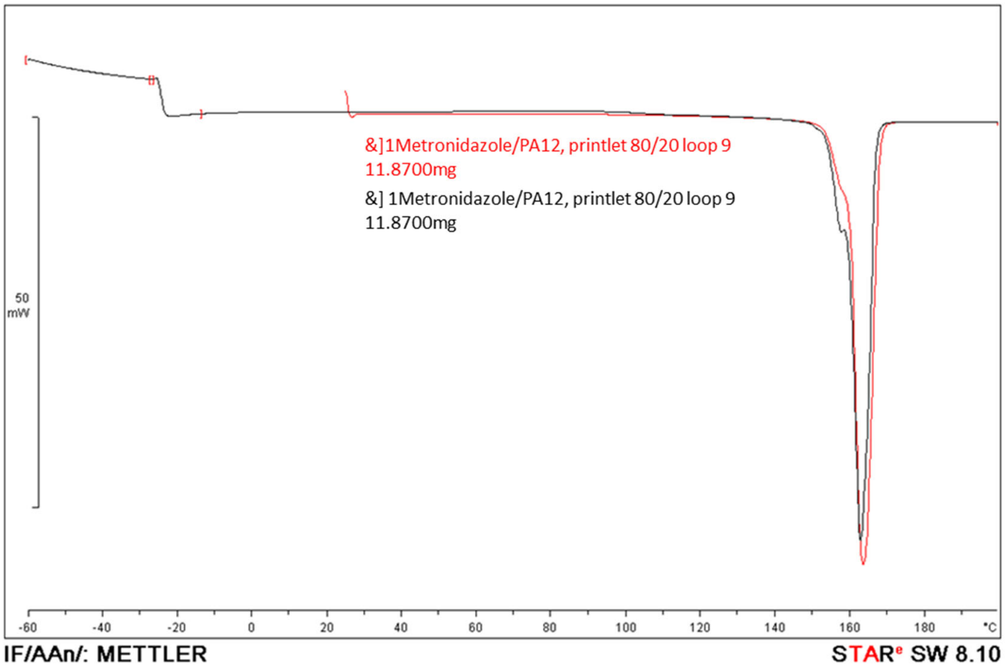

Figure S12. DSC curves from thermal loop of printed tablets, segment 1 and 4 – melting.
